# Sulfated glycosaminoglycans from crown-of-thorns Acanthaster planci – extraction and quantification analysis

**DOI:** 10.1002/fsn3.10

**Published:** 2013-01-08

**Authors:** Nur Afiqah Bahrom, KNS Sirajudeen, George W Yip, Aishah A Latiff, Farid Che Ghazali

**Affiliations:** 1 Biomedicine Programme, School of Health Sciences, Universiti Sains MalaysiaKubang Kerian 16150, Malaysia; 2 Department of Chemical Pathology, School of Medical Sciences, Universiti Sains MalaysiaKubang Kerian 16150, Malaysia; 3 Department of Anatomy, Yong Loo Lin School of Medicine, National University of SingaporeSingapore 117597, Singapore; 4 Doping Control Centre, Universiti Sains MalaysiaPenang 11800, Malaysia

**Keywords:** Acanthaster planci, crown-of-thorns, starfish and GAGs, sulfated glycosaminoglycans

## Abstract

In this article, the novel inventive steps for the extraction and quantification of sulfated glycosaminoglycan (GAG) from *Acanthaster planci* starfish, generally known as crown-of-thorns (COT), are reported. Starfish have been implicated with collagenous distributions within their body anatomy, thus making it a prima facie fact searching for the possibility that GAGs can be isolated from COT. In this study, total-, *N*-, and *O*-sulfated GAGs were extracted from three anatomical regions of the COT (integument, internal tissue, and coelomic fluid) and comparison was made. The result showed that body region of COT seemed to contain higher amount of sulfated GAGs as opposed to the arm region (55.79 ± 0.65 *μ*g/mg was the highest amount in the body extracted from its coelomic fluid and 32.28 ± 3.14 *μ*g/mg was the highest amount in the arm extracted from its internal tissue). COT's integument and coelomic fluid from its body region possessed the highest total of sulfated GAGs content with no significant difference (*P* < 0.05) between the two. All GAGs from COT comprised a higher percentage of *N*-sulfated GAGs than its counterpart, the *O*-sulfated GAGs. When compared with a similar previous study that used sea cucumbers as the sulfated GAGs source, COT possessed more total sulfated GAGs content per milligram as compared with the sea cucumber generally. This result seems to unveil this marine species' advantage per se pertaining to GAGs extraction biomass applicability. Thus, COT could now be the better alternative source for production technology of total-, *N*-, and *O*-sulfated GAGs.

## Introduction

Glycosaminoglycans (GAGs) are series of repeating disaccharide building blocks in an unbranched polysaccharide complex (Esko et al. 2009). Its biochemical building block consists of *N*-substituted amino acids (*N*-acetylglucosamine or *N*-acetylgalactosamine) and a uronic acid (glucoronic acid or iduronic acid) or galactose. There are four classes of glycosaminoglycans: (1) chondroitin sulfate and dermatan sulfate, (2) heparan sulfate and heparin, (3) keratan sulfate, and (4) hyaluronan (Kimata et al. 2007). Hyaluronan seems to be the only glycosaminoglycan without sulfate groups, thus making it the only nonsulfated GAG. With the exception of hyaluronic acid, which exists as a free polymer, all GAGs are covalently attached to core proteins to form proteoglycans (Kjellén and Lindahl 1991). Heparan sulfate and heparin differ from other sulfated GAGs due to the presence of both *O*- and *N*-sulfated groups in their molecule structures (Esko and Lindahl 2001), while the other sulfated GAGs contain only *O*-sulfated group (Sugahara et al. 2003). GAGs are the primary component of the extracellular matrix in animal tissues (Yamada et al. 2011). Anticoagulant and antithrombotic characteristics are among the most widely studied properties of the sulfated polysaccharides (Tovar et al. 2011). Other physiological functions of the GAGs are involved in cell proliferation (Nikitovic et al. 2005), regulators of cell differentiation (Smith et al. 2005), associate in wound healing (Zou et al. 2004; Annika et al. 2007; Masre et al. 2010), endothelial cell receptor for cellular regulation (Rouet 2005), and were also reported to have involved in cancer medicament (Sasisekharan et al. 2002; Lee et al. 2003; Yip et al. 2006; Lamoureux et al 2009; Nikolova et al. 2009).

*Acanthaster planci* (Linnaeus) or crowns-of-thorns (COTs) are starfish categorized under the phylum Echinodermata (Fraser et al. 2000). It was first described by Georg Rumphius in 1705 and was later renamed by Linnaeus in 1758 (Evans 2009). Morphologically, rather than being a typical five-armed starfish, this marine invertebrate can accumulate up to 18 arms extensions when it reaches adulthood. These invertebrates can grow up to 1 m in diameter, but the average size is only about 25–35 cm, with dorsally placed poisonous spines and is the only known poisonous starfish (Sikorski 2006). COTs were found throughout the Indo-Pacific region, ranging from the Red Sea and East Africa of Indian ocean to the Pacific (from mainland Japan south to Lord Howe Island, and from the west coast of Panama to the Gulf of California) (Moran 1988). These COTs are intertidal predators that feed on coral reef vegetations; thus, their existence is worrisome as they aggravate the damage and ultimately leads to total coral reef deforestation. So far, the prompt management of these COTs endemic was to collect and bury these invertebrates in the shore sand bed (Fraser et al. 2000).

GAGs are found not only in vertebrates but also in many invertebrates, implying a conserved presence and function within the animal kingdom (Yamada et al. 2011). Approximately at least 10 phyla are concerned related, of which some of the said invertebrate phyla are Cnidaria, Nematoda, Echinodermata, Annelida, and Mollusca (Medeiros et al. 2000; Yamada et al. 2011). Per se, however, in this study, we will only focus on Echinodermata. There are numerous studies that have proved the exploitable existence of GAGs in Echinoderms. Literatures have documented it to be immensely found in variegated sea cucumber species, such as *Stichopus vastus*, *Stichopus hermanni* (Masre et al. 2011), *Stichopus japonicas* (Kariya et al. 1990), and *Ludwigothurea grisea* (Landeira-Fernandez et al. 2000); sand dollar *Mellita quinquisperforata* (Medeiros et al. 2000); sea urchin *Strongylocentrotus purpuratus* (Yamada et al. 2011) and *Hemicentrotus pulcherrimus* (Fujita et al. 2010); and some other species of Echinoderms. However, up-to-date, extraction of GAGs from COT has never been attempted before, thus leading to poor scientific evidence and research at present to elucidate and note the subsistence of it in COT biomass, especially the one from local coastal region. Hence, in this study, we shall address the scientific lacuna by extracting sulfated GAGs from these Echinoderms and quantify it by its total and divisions of *N*- and *O*-sulfated GAGs components that should be considered a discovery research and an outsource of the alternative source from marine sources, specifically as a sustainable exploitation from wasted COT biomass. This effort should hopefully someday lead to successful large-scale production of GAGs from these Echinoderms that will be beneficial to human health and future production technology.

## Materials and Methods

Fresh samples of COT were harvested from coastal coral reef of Perhentian Island in the state of Terengganu under the supervision of Marine Park Malaysia Department. Voucher specimens were prepared and registered as PPSK/USM/6171139-0611-APLC. These samples were then deposited at the Sea Cucumber Research Center, School of Health Sciences, Universiti Sains Malaysia in Kubang Kerian Health Campus. Selected anatomical tissues of these COTs were then biopsied, and its coelomic fluid was also collected using hypodermic needles. GAGs extraction from the COT was carried out by the method of Ledin et al. (2004) adapted from Staatz et al. (2001), while the quantification analyses were done using Blyscan™ Sulfated Glycosaminoglycan Assay kit (Biocolor, Northern Ireland, U.K.) in accordance with the manufacturer's protocol. Experimental details relating to this study are available as Data S1.

## Results and Discussion

Even though extraction of sulfated GAGs from whole COT anatomical mass has never been attempted before, especially for production technology exploitation, some documented previous microscopic and analytical studies have elucidated that anatomical compartments of COT and other species of starfish do contain collagen (Matsumura 1973; O'Neill 1989; Kimura et al. 1993; Bahrom et al. 2011). Thus, in tandem to this, we hypothesize that GAGs are present. Collagen and GAGs are closely associated as fibrils contained GAGs that are covalently associated with collagen constituents (Trotter et al. 1995). These two are also major components of extracellular matrix, which determines the physical characteristics of tissues and many of the biological properties of cells embedded in it, thus endorsing a dynamic intercellular regulation and communication (Varki et al. 1999).

**Figure 1:**
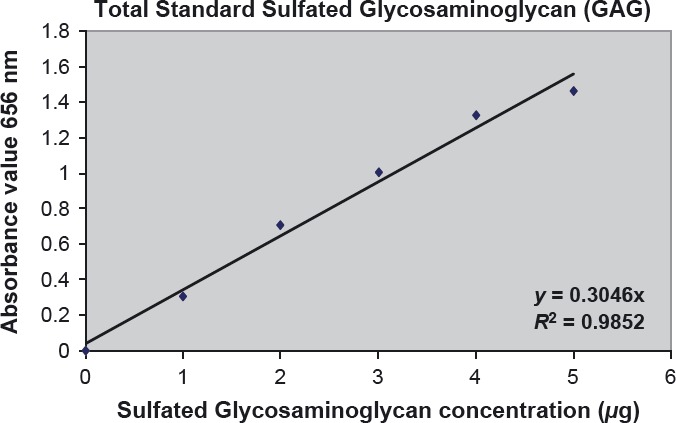
Linear calibration curve using chondroitin 4-sulfate as a standard. This calibration curve was performed by plotting the mean of three sets of standard solution after subtracting the mean duplicate absorbance readings of the reagent blank.

Linearity of the quantification of total sulfated GAG from each anatomical part of COT *A. planci* was ascertained via a standard calibration curve. The standard calibration curve was included into each assay performed, with the appropriate diluted standard solutions of GAG prepared and processed as described in the Materials and Methods section. Figure 1 shows the standard linear curve obtained by plotting the absorbance values of 1,9-dimethylmethylene blue in solution after decomplexation from chondroitin-4 versus its concentration.

**Figure 2:**
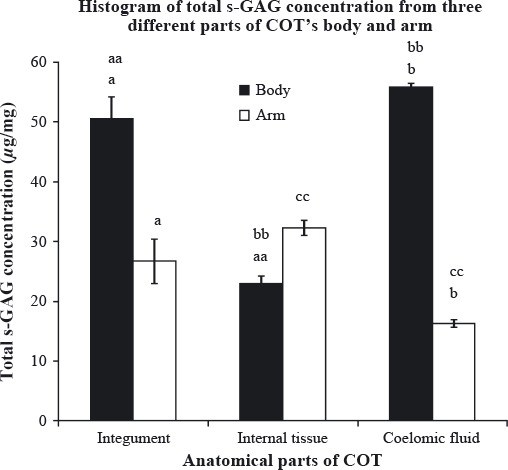
Total sulfated glycosaminoglycan (GAG) content (*μ*g/mg) of three different anatomical parts of crown-of-thorns (COT)'s body and arm. Each analysis was performed in triplicates and data presented in mean ± standard error of mean (SEM). (a,b), significant difference between the same anatomical parts of different region, *P* < 0.05; (aa,bb), significant difference between the anatomical parts from body region, *P* < 0.05; (cc), significant difference between the anatomical parts from arm region, *P* < 0.05.

Figure 2 shows the total sulfated GAG content (*μ*g/mg) of all three anatomical parts (integument, internal tissue, and coelomic fluid) from COT's body and arm. Body's coelomic fluid contained the highest amount of total sulfated GAG (55.79 ± 0.65 *μ*g/mg), followed by body's integument (50.53 ± 3.72 *μ*g/mg), arm's internal tissue (32.28 ± 3.14 *μ*g/mg), arm's integument (26.74 ± 2.75 *μ*g/mg), body's internal tissue (22.98 ± 1.23 *μ*g/mg), and the lowest amount was extracted from arm's coelomic fluid (16.32 ± 1.80 *μ*g/mg). Statistical analysis was done to compare and find any significant differences in total sulfated GAG content of the three anatomical parts within each and between regions using one-way analysis of variance (ANOVA) and independent *t*-test, respectively (Fig. 2).

Independent *t*-test conducted revealed that total sulfated GAG content was significantly different (*P* < 0.05) between integument from body and integument from arm. The result was also similar to coelomic fluid, where significantly higher amount of sulfated GAG was extracted from the body as compared with the arm.

From body region, one-way ANOVA revealed that there were significant differences (*P* < 0.05) between integument and internal tissue; between coelomic fluid and internal tissue, while the arm region showed that there was significant difference (*P* < 0.05) between internal tissue and coelomic fluid. For additional information, there was no significant difference between the body's coelomic fluid, which yielded the highest amount of sulfated GAG, and the body's integument, which was the second highest.

Overall, the body region of the COT contained the highest amount of total sulfated GAG as compared with the arm region, even though the internal tissue from the body has lower amount than its counterpart from the arm region. However, as there is no significant difference in the amount of sulfated GAG between these two regions, we can assume that the body contains higher amount of total sulfated GAG all together than the arm.

**Figure 3:**
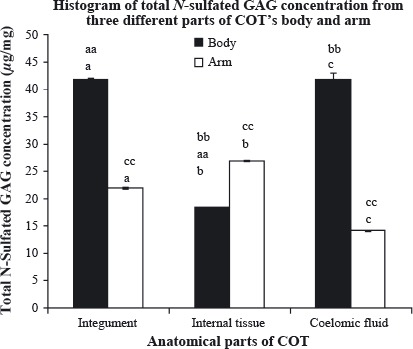
Total *N*-sulfated glycosaminoglycan (GAG) content (*μ*g/mg) of three different anatomical parts of crown-of-thorns (COT)'s body and arm. Each analysis was performed in triplicates and data presented in mean ± standard error of mean (SEM). (a,b,c), significant difference between the same anatomical parts of different region, *P* < 0.05; (aa,bb), significant difference between the anatomical parts from body region, *P* < 0.05; (cc), significant difference between the anatomical parts from arm region, *P* < 0.05.

Figure 3 highlights the amount of total *N*-sulfated GAG from all three anatomical parts of COT's body and arm. The body's integument showed the highest amount of total *N*-sulfated GAG (41.91 ± 0.25 *μ*g/mg), followed closely by the body's coelomic fluid (41.85 ± 1.26 *μ*g/mg), arm's internal tissue (26.95 ± 0.05 *μ*g/mg), arm's integumental wall (21.91 ± 0.20 *μ*g/mg), body's internal tissue (18.39 ± 0.07 *μ*g/mg), while arm's coelomic fluid accounted for the lowest content, which was 14.17 ± 0.13 *μ*g/mg. Further statistical analysis was displayed in Figure 3. Independent *t*-test was carried out to compare the level of total *N*-sulfated GAG between the same anatomical parts from different regions (body and arm). All three anatomical parts showed significant differences (*P* < 0.05) between the body and the arm. One-way ANOVA was carried out to compare the level of total *N*-sulfated GAG between anatomical parts within each region. As for the body, there were significant differences (*P* < 0.05): between integument and internal tissue; between coelomic fluid and internal tissue, while the arm region showed that there were significant differences (*P* < 0.05) in all three anatomical parts.

**Figure 4:**
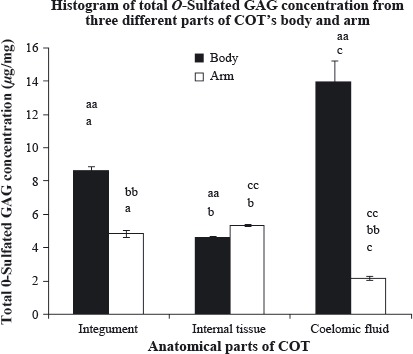
Total *O*-sulfated glycosaminoglycan (GAG) content (*μ*g/mg) of three different anatomical parts of crown-of-thorns (COT)'s body and arm. Each analysis was performed in triplicates and data presented in mean ± standard error of mean (SEM). (a,b,c), significant difference between the same anatomical parts of different region, *P* < 0.05; (aa), significant difference between the anatomical parts from body region, *P* < 0.05; (bb,cc), significant difference between the anatomical parts from arm region, *P* < 0.05.

Table 1 shows the percentage (%) division of *N*- and *O*-sulfated GAGs from its total sulfated GAG content for all three anatomical parts of COT's body and arm. In summary, the total sulfated GAGs were dominated by *N*-sulfated group for all anatomical parts in both COT's regions. This result is congruent with the result found by Regnault and Durrand (1998)_,_ which was researched on crab species, where *N*-sulfated GAGs percentage was higher as compared with *O*-sulfated GAGs percentage. In body region, the highest percentage of *N*-sulfated GAG was possessed by the integument, while the lowest was extracted from the coelomic fluid. However, the coelomic fluid has the highest percentage of *O*-sulfated GAG, while the integument has the lowest. As for the arm region, the result was a complete reversal from the body as the highest percentage of *N*-sulfated GAG was found in coelomic fluid, while the integument contained the lowest percentage. The percentage of the *O*-sulfated GAG from the arm also showed the same pattern of reversal when the highest content was found in the integument instead of within the coelomic fluid.

**Table 1:** Percentage (%) division of N- and O-sulfated glycosaminoglycans (GAGs).

Region	Anatomical parts	*N*-sulfated GAG content (%)	*O*-sulfated GAG content (%)
Body	Integument	82.94 ± 0.50	17.06 ± 0.50
	Internal tissue	80.00 ± 0.30	20.00 ± 0.30
	Coelomic fluid	75.01 ± 2.25	24.99 ± 2.25
Arm	Integument	81.95 ± 0.75	18.05 ± 0.75
	Internal tissue	83.50 ± 0.14	16.50 ± 0.14
	Coelomic fluid	86.86 ± 0.80	13.14 ± 0.80

Except for hyaluronic acid, all GAGs possess *N*- and/or *O*-sulfate groups distributed on their disaccharide building blocks (Barbosa et al. 2003). Based on the results, all GAGs extracted from COT were thus not from the species of hyaluronic acid. Up until now, the presence of hyaluronic acid outside of vertebrates has been reported only in a mollusk of species *Mytilus galloprovincialis*, in detail (Volpi and Maccari 2003).

A study similar to this by Masre et al. (2011 will be compared with the results obtained. Masre et al. who used two kinds of sea cucumber species (*Stichopus vastus* and *Stichopus hermanni*) also analyzed the extracted GAGs quantitatively by its total and *N*- and *O*-sulfated groups distributions. Both sea cucumbers revealed that integumental wall generated the highest content of total sulfated GAGs, while coelomic fluid produced the lowest content. However, our results showed that both integumental wall and coelomic fluid in body region have the highest amount of total sulfated GAGs, as no statistically significant difference between them was observed. COT, however, possessed more total sulfated GAGs content per milligram as compared to sea cucumber generally. The highest amount extracted from COT was 55.79 ± 0.65 *μ*g/mg (from body's coelomic fluid), while *S. vastus*'s integument and *S. hermanii*'s integument yielded 34.97 ± 4.06 and 32.23 ± 6.76 *μ*g/mg, respectively. This unveiled the advantages in terms of applicability that COT will be a better alternative source for biomass production of sulfated GAGs for future biomedical and pharmaceutical purposes as compared with sea cucumbers. Masre et al. (2011 have shown that local sea cucumbers species seem to contain higher percentage of *O*-sulfated GAGs as compared with *N*-sulfated GAGs, per contra with our findings. Per se, the extracted GAGs divisions from COT in this study seems to be contradicting the result by Masre et al. (2011 where COT contains a higher percentage of *N*-sulfated GAGs as compared with *O*-sulfated GAGs percentage. This variation may be due to several factors such as circumstances of temporal climates changes (Black 1954) and limitation of air perceived (Regnault and Durrand 1998).

## Conclusion

In this study, a better yield of sulfated GAGs has been successfully extracted from crown-of-thorns *A. planci*, and the quantitative studies conducted suggest that a sustainable production technology is achievable. The high content of GAGs from the wasted COT biomass can thus be a better tangible source of collagenous threads as compared with other outsourced Echinodermata mass.

## Supporting Information

Data S1 Details methodology.

## References

[b1] Annika NA, Hinke MAB, John RC (2007). Syndecans in wound healing, inflammation and vascular biology. Int. J. Biochem. Cell Biol..

[b2] Bahrom NA, Sirajudeen KNS, Yip GW, Latiff AA, Ghazali FC (2011). Microscopical study of corallivores Crown-of-thorn *Acanthaster planci*. Ann. Microsc..

[b3] Barbosa I, Garcia S, Barbier-Chassefi V, Caruelle JP, Martelly I, Papy-Garcoa D (2003). Improved and simple micro assay for sulfated glycosaminoglycans quantification in biological extracts and its use in skin and muscle tissue studies. Glycobiology.

[b4] Black WAP (1954). Seasonal variation in the combined L-fucose content of the common British Laminariaceae and Fucaceae. J. Sci. Food Agric..

[b5] Esko JD, Lindahl U (2001). Molecular diversity of heparin sulphate. J. Clin. Invest..

[b6] Esko JD, Kimata K, Lindahl U (2009). Proteoglycans and sulfated glycosaminoglycan. Chapter 16 Essentials of glycobiology.

[b7] Evans J (2009). Sea remedies: evolution of the senses.

[b8] Fraser N, Crawford BR, Kusen J (2000). Best practices guide for Crown-of-thorns clean-ups.

[b9] Fujita K, Takechi E, Sakamoto N, Sumiyoshi N, Izumi S, Miyamoto T (2010). HpSulf, a heparan sulfate 6-*O*-endosulfatase, is involved in the regulation of VEGF signaling during sea urchin development. Mech. Dev..

[b10] Kariya Y, Watabe S, Hahimoto K, Yoshida K (1990). Occurrence of chondroitin sulfate E in glycosaminoglycan isolated from the body wall of the sea cucumber *Stichopus japonicas*. Carbohydr. Res..

[b11] Kimata K, Habuchi O, Habuchi H, Watanabe H, Kamerling JP, Boons GJ (2007). Knockout mice and proteoglycans, Comprehensive glycoscience.

[b12] Kimura S, Omura Y, Ishida M, Shirai H (1993). Molecular characterization of fibrillar collagen from the body wall of starfish *Asterias amurensis*. Comp. Biochem. Physiol..

[b13] Kjellén L, Lindahl U (1991). Proteoglycans: structure and interactions. Annu. Rev. Biochem..

[b14] Lamoureux F, Picarda G, Garrigue-Antar G (2009). Glycosaminoglycans as potential regulators of osteoprotegerin therapeutic activity in osteosarcoma. Cancer Res..

[b15] Landeira-Fernandez AM, Aiello KRM, Aquino RS, Silva LCF, deMeis L, Mourao PAS (2000). A sulfated polysaccharide from the sarcoplasmic reticulum of sea cucumber smooth muscle is an endogenous inhibitor of the Ca^2^^+^-ATPase. Glycobiology.

[b16] Ledin J, Staatz W, Li JP, GÖtte M, Selleck S, Kjellén L (2004). Heparan sulfate structure in mice with genetically modified heparan sulfate production. J. Biol. Chem..

[b17] Lee YS, Yang HO, Shin KH, Choi HS, Jung SH, Kim YM (2003). Suppression of tumor growth by a new glycosaminoglycan isolated from the African giant snail *Achatina fulica*. Eur. J. Pharmacol..

[b18] Masre SF, Yip GW, Sirajudeen KNS, Ghazali FC (2010). Wound healing activity of total sulfated glycosaminoglycan (GAG) from *Stichopus vastus**Stichopus hermanni* integumental tissue in rats. Int. J. Mol. Med..

[b19] Masre SF, Yip GW, Sirajudeen KNS, Ghazali FC (2011). Quantitative analysis of sulfated glycosaminoglycans content of Malaysian sea cucumber *Stichopus hermanni**Stichopus vastus*. Nat. Prod. Res..

[b20] Matsumura T (1973). Shape, size and amino acid composition of collagen fibril of the starfish *Asterias amurensis*. Comp. Biochem. Physiol..

[b21] Medeiros GF, Mendes A, Castro RAB, Bau EC, Nader HB, Dietrich CP (2000). Distribution of sulfated glycosaminoglycans in the animal kingdom: widespread occurrence of heparin-like compounds in invertebrates. Biochim. Biophys. Acta.

[b22] Moran P (1988). The *Acanthaster* phenomenon. Aust. Inst. Mar. Sci. Monogr..

[b23] Nikitovic D, Zafiropoulos A, Tzanakakis GN, Karamanos NK, Tsatsakis AM (2005). Effects of glycosaminoglycans on cell proliferation of normal osteoblasts and human osteosarcoma cells depend on their type and fine chemical compositions. Anticancer Res..

[b24] Nikolova V, Koo CY, Ibrahim SA, Wang Z, Spillmann D, Dreier R (2009). Differential roles for membrane-bound and soluble syndecan-1 (CD138) in breast cancer progression. Carcinogenesis.

[b25] O'Neill P (1989). Structure and mechanics of starfish body wall. J. Exp. Biol..

[b26] Regnault M, Durrand F (1998). Glycosaminoglycans in gills of an intertidal crab (*Carcinus maenas*): change in the GAG population in response to prolonged air exposure. Comp. Biochem. Physiol..

[b27] Rouet V (2005). A synthetic glycosaminoglycan mimetic binds vascular endothelial growth factor and modulates angiogenesis. J. Biol. Chem..

[b28] Sasisekharan R, Shriver Z, Venkataraman G, Narayanasami U (2002). Roles of heparan-sulphate glycosaminoglycans in cancer. Nat. Rev. Cancer.

[b29] Sikorski J (2006). Topics in oceanography.

[b30] Smith RA, Meade K, Pickford CE, Holley RJ, Merry CL (2011). Glycosaminoglycans as regulators of stem cell differentiation. Biochem. Soc. Trans..

[b31] Staatz WD, Toyoda H, Kinoshita-Toyoda A, Chhor K, Selleck SB, Iozzo RV (2001). Analysis of proteoglycans and glycosaminoglycans from *Drosophila*, Methods in molecular biology, proteoglycan protocols.

[b32] Sugahara K, Mikami T, Uyama T, Mizuguchi S, Nomura K, Kitagawa H (2003). Recent advances in the structural biology of chondroitin sulphate and dermatan sulphate. Curr. Opin. Struct. Biol..

[b33] Tovar AMF, Teixeira LAC, Pinho DA, Silca LP, Mourao PAS (2011). The dermatan sulfate-dependent anticoagulant pathway is mostly preserved in aneurysm and in severe atherosclerotic lesions while the heparan sulfate pathway is disrupted. Clin. Chim. Acta.

[b34] Trotter JA, Lyons-Levy G, Thurmond FA, Koob TJ (1995). Covalent composition of collagen fibrils from the dermis of the sea cucumber, *Cucumaria frondosa*, a tissue with mutable mechanical properties. Comp. Biochem. Physiol. A.

[b35] Varki A, Cummings R, Esko J, Freeze H, Hart G, Marth J (1999). Proteoglycans and glycosaminoglycans, Essentials of glycobiology.

[b36] Volpi N, Maccari F (2003). Purification and characterization of hyaluronic acid from the mollusc bivalve *Mytilus galloprovincialis*. Biochimie.

[b37] Yamada S, Sugahara K, Ozbek S (2011). Evolution of glycosaminoglycans – comparative biochemical study. Commun. Integr. Biol..

[b38] Yip GW, Smollich M, Gotte M (2006). Therapeutic value of glycosaminoglycans in cancer. Mol. Cancer Ther..

[b39] Zou XH, Foong WC, Cao T, Bay BH, Ouyang HW, Yip GW (2004). Chondroitin sulfate in palatal wound healing. J. Dent. Res..

